# Molecular MRI of Collagen Enables Evaluation of Fibrosis and Therapeutic Response in Venous Thrombosis

**DOI:** 10.1161/CIRCIMAGING.125.018784

**Published:** 2025-12-10

**Authors:** Ling Gao, Nadia Chaher, Joana C. Serralha, Laura Bertolaccini, Carlos Velasco, Gastão Lima da Cruz, Alexander P. Morrell, Claudia Prieto, René M. Botnar, Alberto Smith, Prakash Saha, Alkystis Phinikaridou

**Affiliations:** 1 Research Department of Cardiovascular Imaging, School of Biomedical Engineering and Imaging Sciences (L.G., N.C., C.V., G.L.d.C., C.P., R.M.B., P.K., A.P.), King’s College London, United Kingdom.; 2 School of Cardiovascular and Metabolic Medicine and Science (J.C.S., L.B., A.S.), King’s College London, United Kingdom.; 3 London Metallomics Facility (A.P.M.), King’s College London, United Kingdom.; 4 British Heart Foundation Centre of Research Excellence (R.M.B., A.S., A.P.), King’s College London, United Kingdom.; 5 Department of Radiology, University of Michigan, Ann Arbor (G.L.d.C.).; 6 School of Engineering (C.P., R.M.B.), Pontificia Universidad Católica de Chile, Santiago.; 7 Institute for Biological and Medical Engineering (R.M.B.), Pontificia Universidad Católica de Chile, Santiago.; 8 Millennium Institute for Intelligent Healthcare Engineering, Santiago, Chile (C.P., R.M.B.).

**Keywords:** collagen, fibrosis, magnetic resonance imaging, molecular imaging, venous thrombosis

## Abstract

**BACKGROUND:**

Fibrosis, with accumulation of type I collagen, is a hallmark of postthrombotic change after deep vein thrombosis (DVT), but tools for its direct detection are lacking. Here, we investigate whether molecular magnetic resonance imaging (MRI) using a collagen-specific gadolinium-based probe can detect and measure changes in collagen during thrombus resolution and in response to treatment in a mouse model of DVT.

**METHODS:**

Venous thrombus was induced in the inferior vena cava of BALB/c mice (n=45), and MRI was performed at day 2 (n=3) and weeks 1, 2, and 3 post-surgery using the collagen-specific probe, EP-3533 (10 μmol/kg; n=11–13/group). A subgroup of mice with DVT (n=7) was treated with pravastatin in drinking water (40 mg/kg per day) for 3 weeks post-DVT. Pre- and post-EP-3533 MRI scans were performed. Magnetic resonance venography was used to measure thrombus volume. Inversion recovery T1-weighted images and T1 maps, pre- and post-contrast, were used to calculate the percent change (%) in Δ contrast-to-noise ratio, Δ signal-to-noise ratio, and Δ relaxation rate. Tissues were used for ex vivo analyses.

**RESULTS:**

EP-3533 uptake increased during thrombus organization and resolution, resulting in MRI signal enhancement, with % Δ contrast-to-noise ratio, % Δ signal-to-noise ratio, and % Δ relaxation rate peaking at 3 weeks after DVT. MRI measurements of collagen accumulation quantified as an increase in % Δ contrast-to-noise ratio (ρ=0.89; *P*=0.012) and % Δ relaxation rate (ρ=0.80; *P*=0.029) correlated positively with collagen histology. The spatial distribution of gadolinium in the tissue colocalized with collagen type I based on immunohistochemistry (ρ=0.95; *P*<0.001). Statin treatment decreased both collagen accumulation and vein wall thickness, without affecting thrombus size.

**CONCLUSIONS:**

Molecular MRI using a collagen-targeting probe made collagenous thrombus visible on MRI and detected changes in collagen content during thrombus resolution.

**CLINICAL PERSPECTIVE**

Current clinical imaging approaches used to diagnose deep vein thrombosis and guide interventions rely on anatomic and blood flow information but do not directly inform on the collagen content of the thrombus and the venous wall that affects treatment outcomes. This study demonstrates that molecular magnetic resonance imaging of collagen allows detection of the collagen content of venous thrombus during its resolution noninvasively. This enables staging of disease progression and the identification of postthrombotic collagenous thrombi. Molecular imaging of collagen provides a novel tool that may be valuable in preclinical drug development, as well as in the diagnosis, therapeutic management, and monitoring of treatment response in patients with deep vein thrombosis.

Deep vein thrombosis (DVT) is a significant cause of morbidity and mortality worldwide^1^ and can lead to serious complications such as pulmonary embolism and postthrombotic syndrome. Current DVT diagnostic assessments include ultrasonography,^2^ magnetic resonance venography, and computed tomography angiography^3^ that detect the location and size of the thrombus. However, these modalities have certain limitations as they do not provide information on thrombus composition, which can affect clinical decision-making.^3,4^

Thrombus organization and resolution are dynamic processes that involve the accumulation of inflammatory cells and the replacement of fibrin with collagen.^4,5^ Studies show that both type I and III collagens increase overtime,^6^ with collagen type I being the most dominant component.^7^ Collagenous thrombus is more resistant to plasmin-mediated degradation,^8–12^ and the extent of collagen may affect outcomes when interventions are being considered.^11,12^ Direct targeting of thrombus components using molecular imaging probes could, therefore, be beneficial.

Small molecular weight–targeted imaging probes have, to date, enabled the detection of specific and abundant components of the thrombus including platelets^13^ and fibrin,^14,15^ as well as monitoring the response to thrombolytic treatment^16^ using magnetic resonance imaging (MRI), positron emission tomography (PET), and single-photon emission computed tomography. Many MRI protocols (including T1 mapping, magnetization transfer, and diffusion-weighted imaging)^4,17^ have also been developed to characterize the composition of venous thrombus without the use of exogenous agents to detect thrombi amendable to thrombolysis. However, a method that selectively detects collagenous/chronic thrombi and differentiates them from fibrin or erythrocyte-rich thrombi with high sensitivity and specificity is lacking. EP-3533, a gadolinium-based MRI contrast agent with high affinity for type I collagen (Kd=1.8 µM), consists of a 10-amino-acid cyclic peptide conjugated to 3 gadolinium (Gd)-diethylenetriamine pentaacetic acid moieties (about 5 kDa).^18,19^ It has been validated preclinically for the detection of fibrosis in the myocardium,^19^ liver,^20^ lung,^21^ and pancreas,^22^ with MRI quantification correlating strongly with histological measurements.

Here, we investigate whether molecular MRI using the collagen-specific gadolinium-based probe, EP-3533, provides insights into venous thrombus collagen composition during its resolution and in response to an intervention using a mouse model of DVT.

## Methods

The data, methods, and study material that support the findings of this study are available from the corresponding author upon reasonable request. All animal procedures were performed in accordance with the guidelines of the UK Home Office Animal (Scientific Procedures) Act 1986, under project license number PP8419036, and conform to the guidelines from Directive 2010/63/EU of the European Parliament on the protection of animals used for scientific purposes. Ethical approval was granted by King’s College London’s Animal Welfare and Ethical Review Body.

### Study Design

This project used a mouse model of DVT, in vivo molecular MRI, and ex vivo tissue analyses. Venous thrombus was induced in the inferior vena cava (IVC) of male BALB/c mice (n=45; 8–10 weeks) through a combination of 80% to 90% stenosis of the IVC and the application of a surgical clip to induce endothelial damage.^4^ Following DVT, mice were allocated either to a progression arm lasting up to 3 weeks (untreated group; n=38) or to a treatment arm receiving statin in drinking water (pravastatin 40 mg/kg per day) for 3 weeks post-DVT (n=7). Mice were imaged using a clinical 3T MRI scanner pre-intravenous injection and 1 hour post-intravenous injection of the collagen-specific contrast agent (EP-3533; 10 μmol/kg). Untreated mice were imaged at day 2 (n=3), week 1 (n=11), week 2 (n=11), and week 3 (n=13) after induction of DVT. Pravastatin-treated mice were imaged at week 3 post-DVT. Magnetic resonance venography was used to calculate the thrombus volume (Figure S1). Precontrast and postcontrast inversion recovery T1-weighted (IR-T1w) images were used to analyze the contrast-to-noise ratio (CNR) and signal-to-noise ratio (SNR). T1 maps were used to measure the relaxation rate (R1=1/T1). % ΔCNR, % ΔSNR, and % ΔR1 were calculated as ([Measurement_postcontrast_−Measurement_precontrast_]/Measurement_precontrast_)*100. After imaging, mice were euthanized using isoflurane inhalation at a concentration of 3% to 4% followed by cardiac puncture. The IVC containing the thrombus and the adjacent aorta were collected, and thrombus volume and collagen content were measured using Masson trichrome and Picrosirius red staining (n=3–5/time point). Additional immunohistochemistry of collagen I and complementary Western blotting (n=3/time point) were performed to semiquantify the tissues’ collagen content. Laser-ablation inductively coupled plasma mass spectrometry was used to map the spatial distribution of gadolinium (and, thus, the collagen-specific probe) on the tissue and quantify the gadolinium concentration (n=3/timepoint). Detailed methods are described in the Supplemental Material.

### Statistical Analysis

Data were analyzed with GraphPad Prism 10 (GraphPad Software, Inc, La Jolla, CA). Normality was assessed by the Shapiro-Wilk test. A 1-way ANOVA followed by the Bonferroni post hoc tests was used to compare across multiple groups for normally distributed variables (eg, thrombus volume, % ΔSNR, and collagen volume based on ex vivo histology). The Student *t* tests were used to compare the untreated and stain-treated groups for normally distributed variables (eg, thrombus volume, % ΔSNR, and collagen volume based on ex vivo histology). For nonnormally distributed data, a Mann-Whitney *U* test or a Kruskal-Wallis test followed by the Dunn tests was used for 2 or multiple groups comparisons analysis (eg, % ΔCNR and % ΔR1). Wilcoxon matched-pairs signed rank tests were performed to analyze precontrast and postcontrast CNR, SNR, and T1 relaxation time post-DVT. Correlation analysis was performed using the Spearman test for the correlation between collagen volume and % ΔCNR. Pearson tests were applied to evaluate the correlation between collagen volume and % ΔR1, and the relation between collagen I area and Gd concentration. Receiver operating characteristic curve analysis was performed to evaluate the sensitivity and specificity of ΔR1 from molecular MRI for detecting collagenous thrombus. *P*<0.05 was considered statistically significant. All the data are presented as mean±SD.

## Results

### In Vivo EP-3533–Enhanced MR Images Detect Collagen Accumulation After DVT

**Figure 1.**
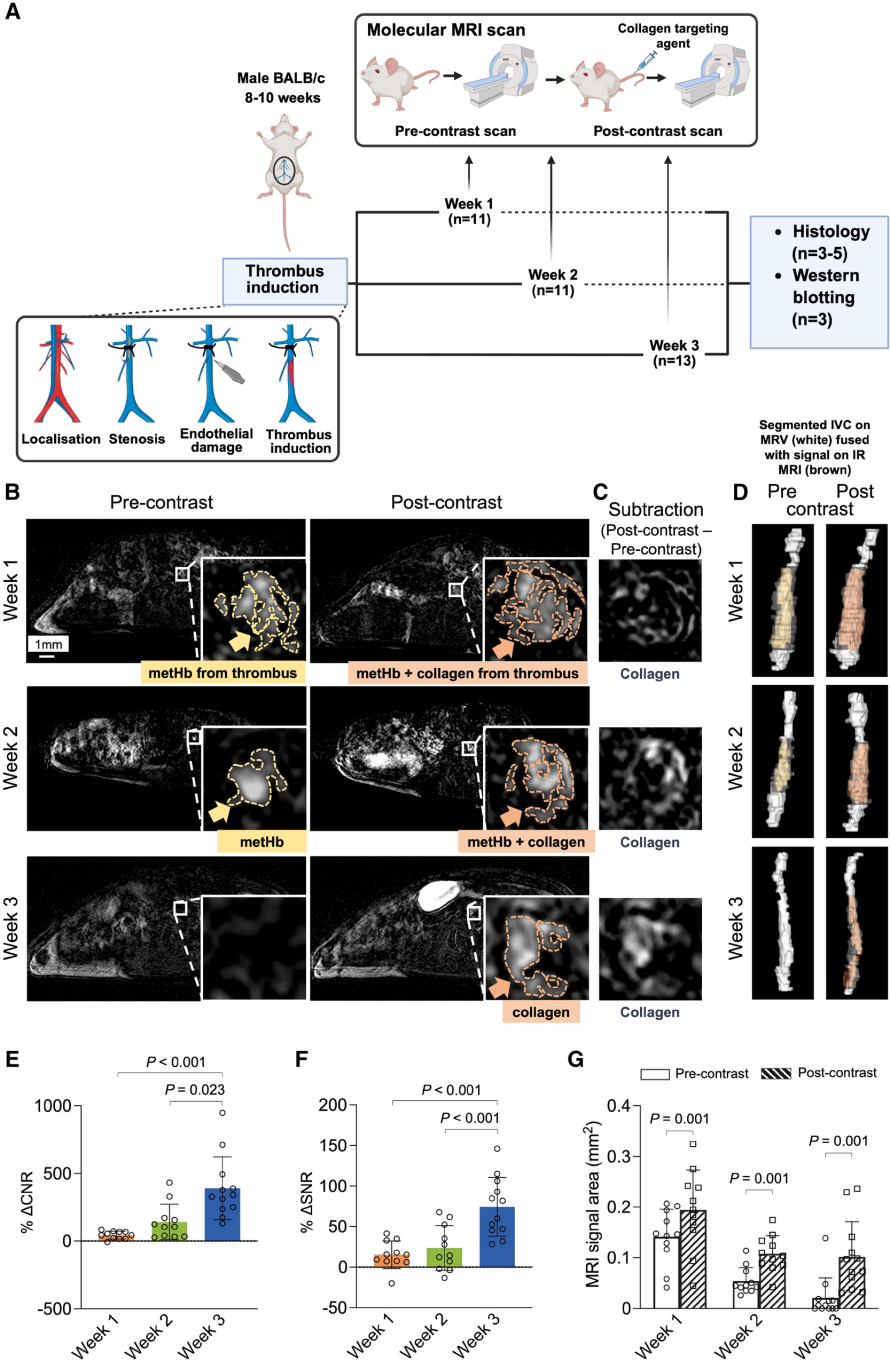
**In vivo EP-3533–enhanced magnetic resonance (MR) images detect collagen accumulation in a mouse model of deep vein thrombosis (DVT). A**, Schematic of the animal model to induce DVT in the inferior vena cava (IVC) and study design. **B**, Representative inversion recovery T1-weighted images, pre- and post-contrast at weeks 1, 2, and 3 post-DVT. Precontrast images show signal at weeks 1 and 2 post-DVT because of metHb (methemoglobin). Uptake of the collagen probe (EP-3533) increases signal enhancement during thrombus organization and resolution, and enables detection of collagenous thrombus, which is invisible on precontrast images at week 3. Light yellow dashed circles indicate the MR imaging (MRI) signal generated from metHb, and light orange dashed circles indicate the MRI signal generated from metHb and collagen. **C**, Subtracted precontrast from postcontrast inversion recovery T1-weighted (IR-T1w) images shows the deposition of collagen within the thrombus detected using the collagen probe. **D**, Fusion of the segmented IVC based on MR venography (MRV; white) and the MRI signal observed on precontrast and postcontrast images after DVT (light brown) show collagen deposition within regions of metHb at weeks 1 and 2 and purely collagenous thrombus at week 3, which are detected only postcontrast. **E** and **F**, Quantification of the % Δ contrast-to-noise ratio (CNR), % Δ signal-to-noise ratio (SNR) of thrombus area across groups. **G**, Quantification of the MRI signal area before and after contrast administration. % ΔCNR and % ΔSNR were calculated as ([Measurement_postcontrast_−Measurement_precontrast_]/Measurement_precontrast_)*100. Data are presented as mean±SD. Kruskal-Wallis followed by the Dunn multiple comparisons test was used for % ΔCNR, and 1-way ANOVA followed by the Bonferroni post hoc test was used for multiple comparisons for % ΔSNR, and the Wilcoxon matched-pairs signed rank test was used for MRI signal area; n=11 for week 1, n=11 for week 2, and n=13 for week 3.

The study design is illustrated in Figure 1A. Due to the oxidation of Fe3^+^ released from erythrocytes during thrombus resolution, the accumulation of paramagnetic metHb (methemoglobin) generates a native signal in the thrombus that is observed on precontrast IR-T1w images.^4,23,24^ In this study, mice were imaged before and after contrast administration to decouple the native signal from that generated after the uptake of EP-3533. In mice with DVT, precontrast IR-T1w images showed a bright signal in the thrombus at weeks 1 and 2, originating from metHb, but no signal at week 3 post-DVT (Figure 1B) when metHb is cleared. Postcontrast IR-T1w images showed selective enhancement within the thrombus and venous wall over time after DVT. Importantly, postcontrast images acquired 3 weeks post-DVT enable detection of collagenous thrombi that were invisible on the precontrast images (Figure 1B and 1C). Quantitative analysis showed increased CNR and SNR following EP-3533 administration, with the most evident increase observed at 3 weeks post-DVT (Figure S2). The IVC, observed on the magnetic resonance venography, and the signal observed on the pre- and post-IR-T1W images were segmented and fused (Figure 1D). The fused images show that the collagen detected on postcontrast IR-T1w images colocalized with the signal from metHb, observed on precontrast images at weeks 1 and 2. At week 3, the postcontrast enhanced images demonstrate the accumulation of collagen along the length of the thrombus that was undetectable on the precontrast IR-T1w images (Figure 1D).

In vivo MR venography and angiography images were used to localize the thrombus and quantify changes in thrombus volume over time. As expected, MRI showed a decrease in thrombus size over time, concurrent with thrombus resolution, from week 1 to week 3 post-DVT (volume: week 1, 13.19±3.64 mm^3^; week 2, 8.32±3.31 mm^3^; and week 3, 3.49±1.58 mm^3^; *P*<0.001; Figure S3). To account for the native signal observed in the precontrast images, the % ΔCNR and % ΔSNR were used to assess the uptake of the collagen probe over time. The % ΔCNR and % ΔSNR, between precontrast and postcontrast enhanced images, were significantly higher at 3 weeks post-DVT, suggesting that these semiquantitative measurements detect temporal changes in collagen content (Figure 1E and 1F; % ΔCNR: week 1, 43.25±28.52%; week 2, 140.90±131.10%; and week 3, 390.50±232.80%; *P*<0.001; % ΔSNR: week 1, 15.25±16.92%; week 2, 23.62±27.55%; and week 3, 74.30±36.41%; *P*<0.001). Quantification of the MRI area, visible on the precontrast and postcontrast enhanced images, was highest at 3 weeks because collagenous thrombi, invisible on the precontrast images, became detectable only after administration of EP-3533 (Figure 1G; Table S1). To further establish the in vivo specificity of the collagen-targeted agent, mice were imaged at day 2 post-DVT when no collagen is present within the thrombus. At this acute phase, no unspecific binding or retention of EP-3533 was observed following administration of EP-3533 (Figure S4).

### T1 Mapping Quantifies EP-3533 Probe Uptake That Increases After DVT

**Figure 2.**
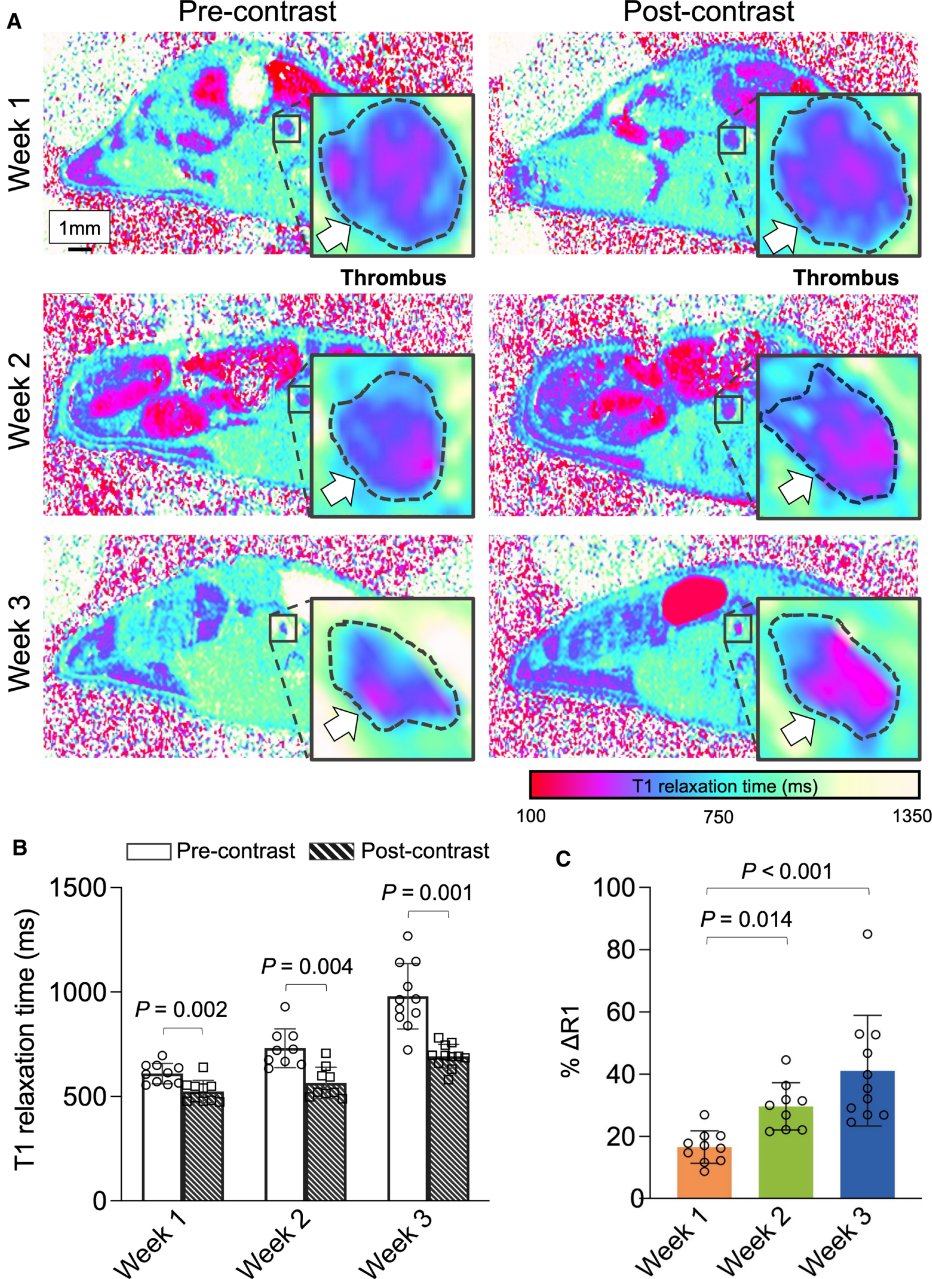
**In vivo T1 mapping quantifies probe uptake and detects increasing collagen deposition after deep vein thrombosis (DVT). A**, Corresponding precontrast and postcontrast enhanced T1 maps after 1, 2, and 3 weeks of DVT. Black dashed circles indicate inferior vena cava (IVC)/thrombus. **B**, Quantification of precontrast and postcontrast T1 relaxation time. **C**, Quantification of the % Δ relaxation rate (R1). % ΔR1 was calculated as ([Measurement_postcontrast_−Measurement_precontrast_]/Measurement_precontrast_)0*100. Data are presented as mean±SD. The Wilcoxon matched-pairs signed rank test was used for T1 relaxation time, and Kruskal-Wallis followed by the Dunn multiple comparisons test was used for % ΔR1; n=10 for week 1, n=9 for week 2, and n=11 for week 3.

Precontrast quantitative T1 mapping showed lower T1 relaxation times at weeks 1 and 2 (because of metHb) and an increase in T1 relaxation time at week 3 (Figure 2A and 2B). Administration of the EP-3533 further reduced the T1 relaxation times at weeks 1, 2, and 3 with the largest decrease observed at 3 weeks post-DVT (Figure 2B; Table S1). Consequently, the % ΔR1 (1/T1) was highest 3 weeks after DVT (week 1, 16.63±5.20%; week 2, 29.70±7.59%; and week 3, 41.15±17.81%; *P*<0.001; Figure 2C), indicative of higher contrast uptake and increased collagen deposition at this time point. In contrast to the observations at weeks 1, 2, and 3 post-DVT, no changes in the T1 or R1 values were observed between precontrast and postcontrast images in acute thrombi at day 2 (Figure S4).

### Detection of Collagen Accumulation After DVT Using In Vivo EP-3533–Enhanced MRI Correlates With Ex Vivo Tissue Analyses

**Figure 3.**
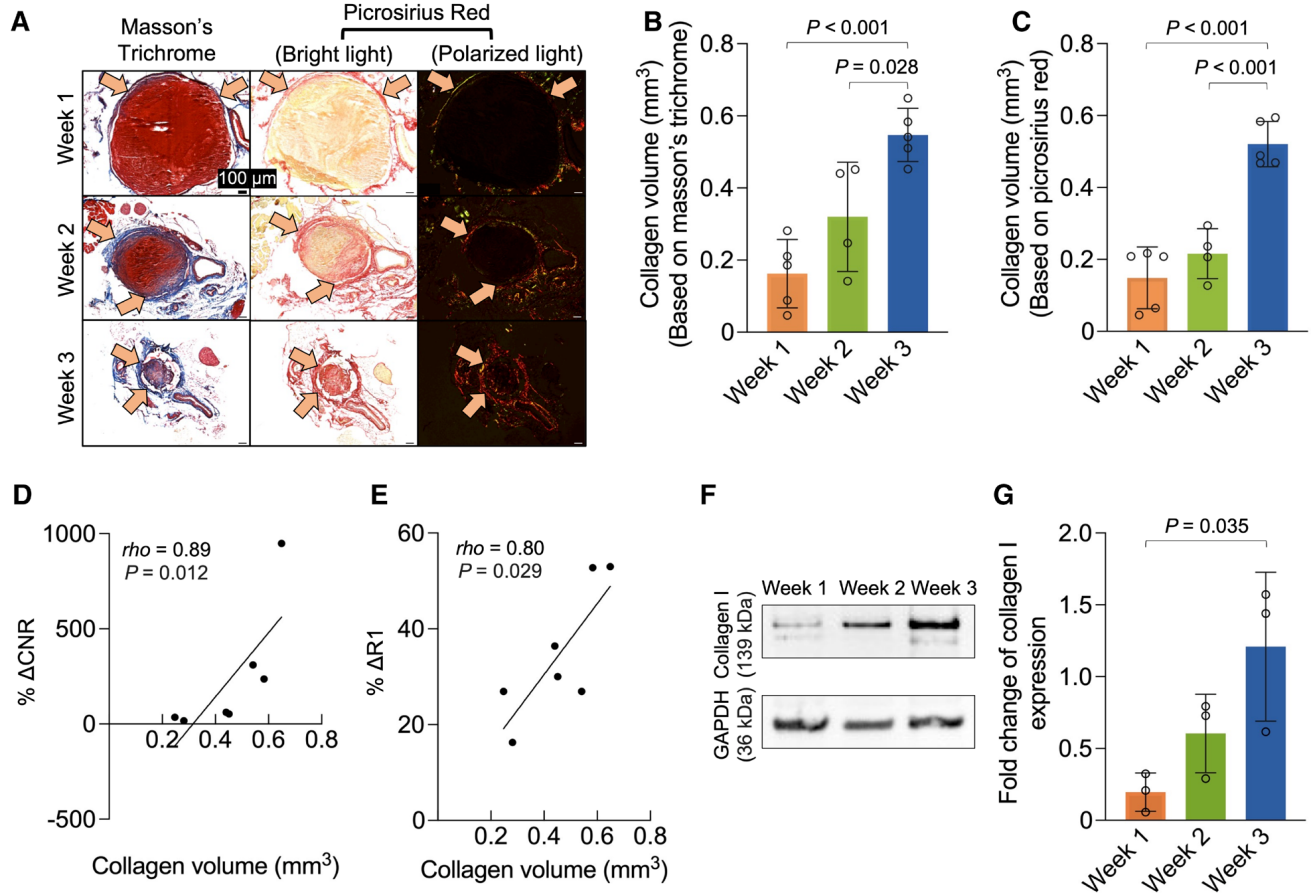
**Tissue analyses of collagen content and correlation with molecular magnetic resonance imaging (MRI). A**, Masson trichrome and Picrosirius red staining of the thrombus and inferior vena cava (n=5 for week 1, n=4 for week 2, and n=5 for week 3; scale bar, 100 µm). **B**, Quantification of collagen volume based on Masson trichrome staining. **C**, Quantification of collagen volume based on Picrosirius red staining viewed under polarized light. **D**, Correlation between the % Δ contrast-to-noise ratio (CNR) and the collagen volume measure by histology. **E**, Correlation between the % Δ relaxation rate (R1) relaxation rate and the collagen volume measured by histology. **F** and **G**, Collagen content measured by the Western blotting (n=3/time point). Orange arrows indicate the collagen deposition. Data are presented as mean±SD. One-way ANOVA followed by the Bonferroni post hoc test was used for multiple comparisons for collagen volume and fold change of collagen I expression, and the Spearman ρ test was used for correlation analysis between collagen volume and % ΔCNR. The Pearson test was used for correlation analysis between collagen volume and % ΔR1.

Masson trichrome and Picrosirius red staining of the IVC containing the thrombus revealed that collagen deposition increased from 1 to 3 weeks post-DVT (Figure 3A). The spatial distribution of collagen observed on histology correlated with regions of signal enhancement observed on EP-3533-enhanced MRI (Figure 1). Histology showed increased collagen content during thrombus organization and resolution (Figure 3B and 3C; Figure S5; Table S2). Importantly, in vivo MRI measures of collagen quantified as % ΔCNR (ρ=0.89; *P*=0.012) and % ΔR1 (ρ=0.80; *P*=0.029) correlated positively with the collagen content measured by histology (Figure 3D and 3E). Receiver operating characteristic curve analysis has also demonstrated that ΔR1 values from molecular MRI detected collagenous thrombi with a sensitivity of 75% and a specificity of 100% (Figure S6). Western blotting also confirmed the accumulation of collagen I during thrombus organization and resolution (Figure 3F and 3G; Table S2).

### The Spatial Distribution and Concentration of EP-3533 Colocalize and Correlate With Collagen

**Figure 4.**
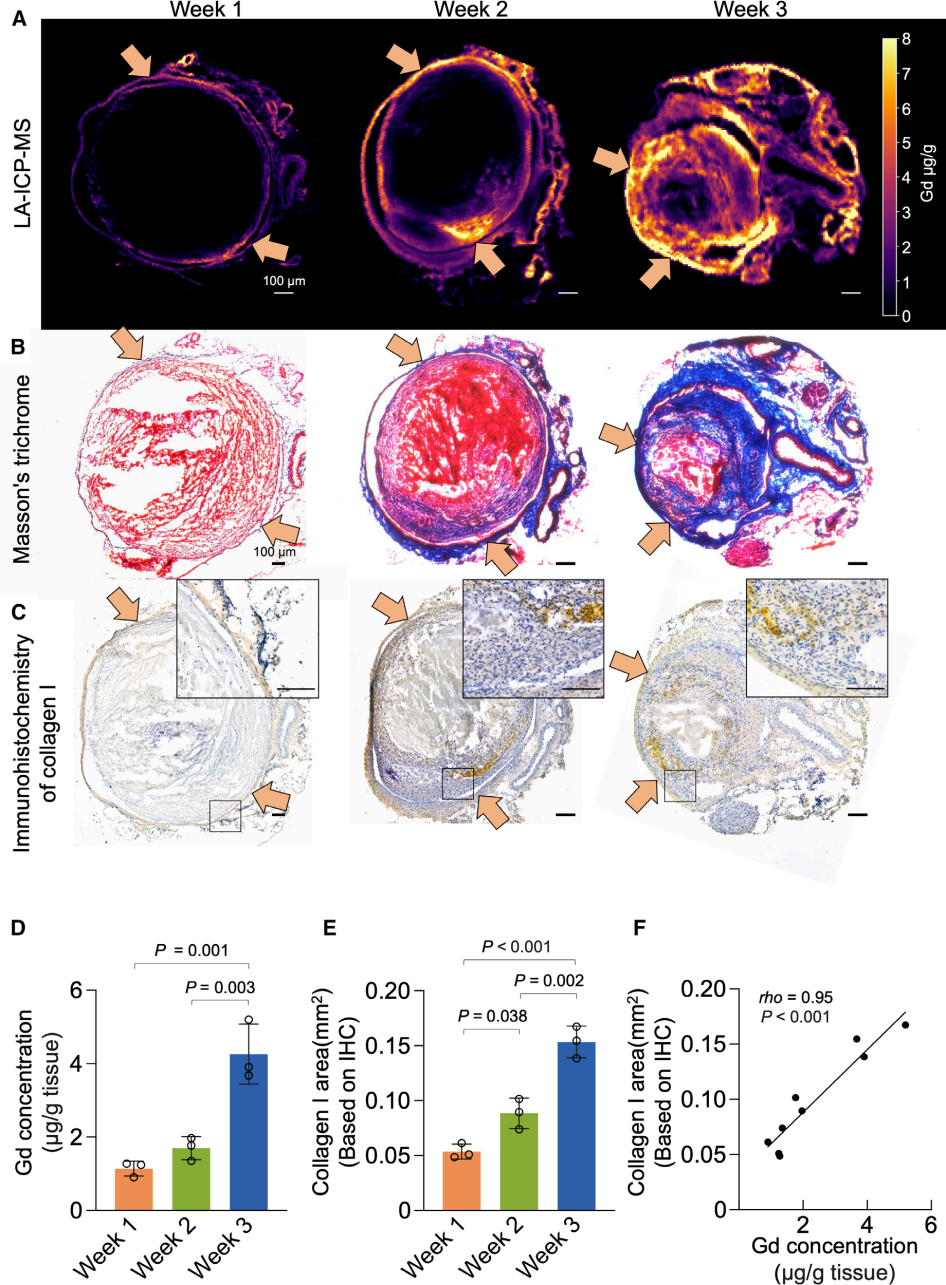
**Spatial distribution of the collagen-specific gadolinium-based probe and quantification of gadolinium concentration in tissues using laser-ablation inductively coupled plasma mass spectrometry (LA-ICP-MS). A**, LA-ICP-MS images show gadolinium (from the collagen-targeting probe) distribution in the vein wall and thrombus. The concentration of gadolinium in the tissue increases during thrombus organization and resolution. Orange arrows indicate the gadolinium distribution. **B** and **C**, Corresponding Masson trichrome staining and immunohistochemistry of collagen I. Orange arrows indicate the collagen deposition. The distribution of gadolinium colocalizes with collagen I fibers (magnified image; scale bar, 100 µm). **D**, Quantification of gadolinium (Gd) concentration in the inferior vena cava (IVC)/thrombus. **E**, Quantification of collagen I area in the IVC/thrombus. **F**, Correlation between Gd concentration and collagen I expression. Data are presented as mean±SD. One-way ANOVA followed by the Bonferroni post hoc test was used for multiple comparisons; n=3/time point. The Pearson test was used for correlation analysis between collagen area and Gd concentration.

To better visualize the distribution and quantify the concentration of EP-3533 in the thrombus and venous wall, sections from weeks 1, 2, and 3 post-DVT were imaged using laser-ablation inductively coupled plasma mass spectrometry. Similar to the in vivo MRI and histological data, laser-ablation inductively coupled plasma mass spectrometry showed that the spatial distribution of the gadolinium (from the collagen-specific probe) extended from week 1 to week 3 post-DVT (Figure 4A). Furthermore, corresponding histology showed colocalization of the gadolinium with collagen, as detected by Masson trichrome (Figure 4B), and more specifically with type I collagen based on immunohistochemistry (Figure 4C). Quantification of gadolinium concentration in the thrombus and venous wall increased from week 1 to week 3 post-DVT (week 1, 1.14±0.20 µg/g; week 2, 1.70±0.31 µg/g; and week 3, 4.26±0.82 µg/g; *P*<0.001; Figure 4D). The deposition of collagen type I fibers was also increased from 1 to 3 weeks post-DVT based on immunohistochemistry (Figure 4E; Table S2). The gadolinium concentration in the tissue positively correlated with the collagen I content based on immunohistochemistry (ρ=0.95; *P*<0.001; Figure 4F).

### Molecular Imaging of Collagen Enables Monitoring of the Antifibrotic Effects of Statins After DVT

**Figure 5.**
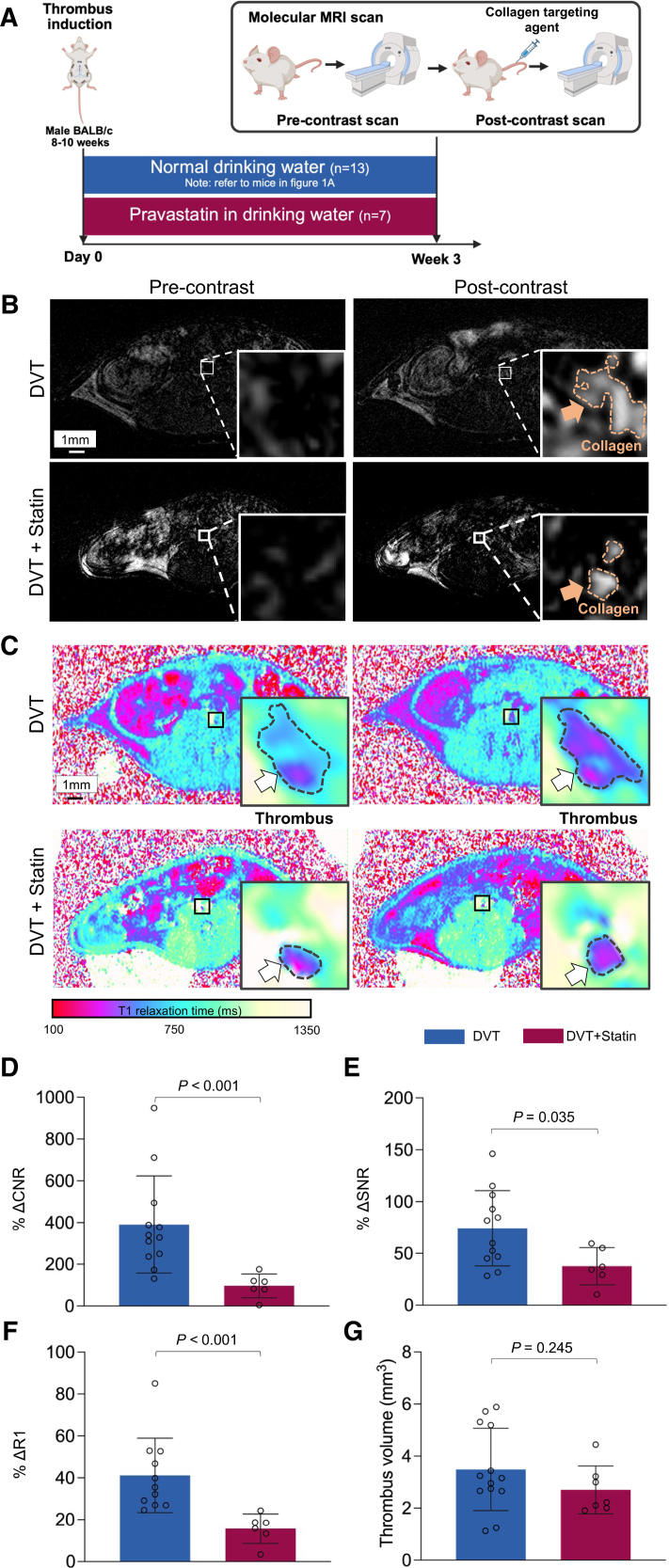
**EP-3533-enhanced magnetic resonance imaging (MRI) enables monitoring of the antifibrotic effects of statins in deep vein thrombosis (DVT). A**, Experimental design. The DVT group refers to animals presented in Figure 1A. **B** and **C**, Precontrast and postcontrast enhanced inversion recovery T1-weighted (IR-T1w) images and T1 maps in untreated and statin-treated mice with DVT. Light orange dashed circles indicate MRI signal generated from the detection of collagen, and black dashed circles indicate inferior vena cava (IVC)/thrombus. Collagenous thrombus at 3 weeks after DVT is invisible on precontrast images but is detected on postcontrast images. Postcontrast images show less enhancement in statin-treated animals. Concurrently, T1 maps show less contrast uptake in mice with DVT treated with statin. **D** through **F**, Quantification of % Δ contrast-to-noise ratio (CNR), % Δ signal-to-noise ratio (SNR), and % Δ relaxation rate (R1) confirms the lower uptake of the collagen-targeting probe in statin-treated mice. **G**, Segmentation of magnetic resonance venography (MRV) images shows similar thrombus volume in untreated and treated mice. Data are presented as mean±SD. % ΔCNR, % ΔSNR, and % ΔR1 were calculated as ([Measurement_postcontrast_−Measurement_precontrast_]/Measurement_precontrast_)*100. The Student *t* test was used for thrombus volume and % ΔSNR, and the Mann-Whitney *U* test was used for thrombus volume and % ΔCNR and % ΔR1 analysis; n=13 for untreated and n=7 for statin-treated mice for in vivo MRI/time point, and n=4 to 5 for histology/time point.

**Figure 6.**
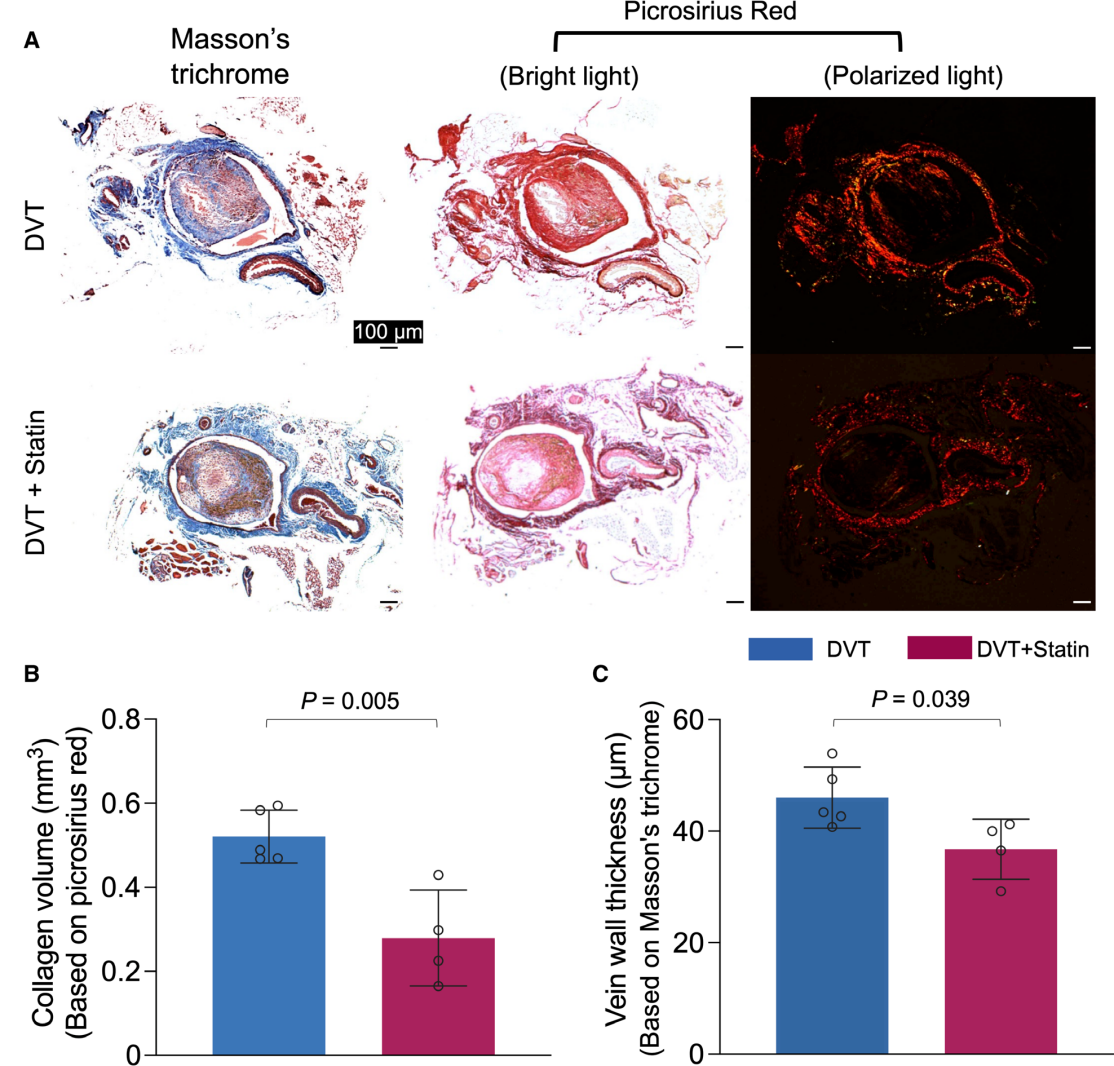
**Ex vivo histological analyses validated the reduction of collagen content in statin-treated mice. A**, Representative Masson trichrome and Picrosirius red stained images of the thrombus in untreated and statin-treated mice (n=5 for untreated and n=4 for statin-treated mice; scale bar, 100 µm). **B**, Quantification of the collagen volume based on the histology shows lower collagen content in statin-treated animals. **C**, The vein wall thickness is reduced in statin-treated mice. Data are presented as mean±SD. The Student *t* test was used for collagen volume and vein wall thickness. DVT indicates deep vein thrombosis.

After demonstrating that in vivo EP-3533-enhanced MRI can detect collagen accumulation following DVT, we next evaluated whether this approach can detect changes in collagen after treatment. Mice with DVT treated with pravastatin for 3 weeks post-DVT were compared with the untreated mice at the same time point (Figure 5A). Postcontrast enhanced images showed less signal enhancement, and T1 maps showed longer T1 relaxation times in the thrombus and venous wall of statin-treated compared with untreated mice (Figure 5B and 5C). Quantification of probe uptake showed lower % ΔCNR, % ΔSNR, and % ΔR1 in statin-treated compared with untreated mice (*P*_% _Δ_CNR_<0.001; *P*_% _Δ_SNR_=0.035; *P*_% _Δ_R1_<0.001; Figure 5D through 5F). Despite changes in the collagen content, quantification of thrombus volume measured by magnetic resonance venography was similar between the 2 groups (DVT, 3.49±1.58 mm^3^ versus DVT+statin, 2.70±0.92 mm^3^; *P*=0.245; Figure 5G). The imaging data were validated by histology showing ≈50% reduction in collagen content in statin-treated mice compared with untreated mice (DVT, 0.52±0.06 mm^3^ versus DVT+statin, 0.28±0.11mm^3^; *P*=0.005; Figure 6A and 6B). Finally, histological analysis showed that statin treatment decreased the thickness of the venous wall (DVT, 46.02±5.47 µm versus DVT+statin, 36.76±5.39 µm; *P*=0.039; Figure 6C).

## Discussion

The aim of this study was to assess the feasibility and potential of molecular MRI to detect and measure changes in collagen content during venous thrombus resolution and in response to a treatment. Using a mouse model of DVT, we demonstrated that the MR probe EP-3533, targeting collagen, detects and quantifies dynamic changes in collagen accumulation during thrombus organization and resolution. Importantly, the use of the molecular probe enabled the detection of collagenous/chronic thrombi that would otherwise evade detection by standard MRI methods. Our findings are the first to show that molecular MRI, using a clinically relevant magnetic field strength, enables direct detection of changes in the collagen composition of venous thrombus. An imaging method that is informative of thrombus age and composition could serve as a valuable tool to enhance diagnosis, guide treatment more effectively, and monitor treatment response in DVT.

In this study, we demonstrate that the uptake of the collagen probe resulted in increased signal enhancement and higher R1 over time, peaking at 3 weeks post-DVT. The probe’s age-dependent uptake and the regions of elevated MRI signal observed in vivo colocalized with sites of collagen accumulation confirmed by ex vivo tissue histology, immunohistochemistry, and Western blotting. Finally, tissue elemental analysis demonstrated that the spatial distribution of the gadolinium collagen probe colocalized both with sites of MRI signal enhancement and with the distribution of collagen I as detected by immunohistochemistry. Our results in the mouse model of DVT are in concordance with and extend previous findings in animal models, showing that EP-3533-enhanced MRI detects fibrotic changes in the heart,^19^ liver,^20^ cancer,^25^ and lungs.^26^

In our study, we also investigated whether collagen-targeted MRI is sensitive to monitor treatment response. Our data showed that statin treatment reduced the collagen content in both the thrombus and venous wall, without affecting the volume of the thrombus. Although statins are not routinely prescribed for patients with DVT, clinical trials have shown that statins exert protective effects against venous thromboembolism.^27,28^ The benefits of statins may arise from pleiotropic effects, such as anti-inflammatory properties, improvement of endothelial function, and reduction of oxidative stress.^29^ Our observation that statins decreased fibrosis in DVT is supported by studies, showing that statins inhibit profibrotic genes (such as *CTGF*, *ACTA2*, and *COL1A1*)^30^ and reduce collagen production by suppressing the TGF-β (transforming growth factor-beta)/Smad (Suppressor of Mothers against decapentaplegic) pathway.^30–32^ Our histological analyses also showed that treatment with a statin reduced vein wall thickness following thrombosis. This observation is in line with reports showing a ≈50% decrease in neutrophil influx and reduction of vein wall thickening in statin-treated mice.^33^ Although the precise molecular and cellular effects of statins on thrombus fibrosis were not explored, our findings suggest that molecular imaging of collagen can sensitively detect changes in collagen content, even when using a nonspecific antifibrotic therapy such as statins.

There have been continued efforts to develop imaging strategies for characterizing thrombus composition and specifically collagenous, chronic thrombus using noninvasive techniques. Ultrasound elastography has shown promise to stage thrombus ex vivo,^34^ but its feasibility in vivo still requires further investigation. Molecular imaging of fibrin using MRI or PET probes has significantly improved thrombus characterization with the goal of identifying those thrombi, which are more likely to lyse.^16,35,36^ Changes in T1 and T2 relaxation time, magnetization transfer, and diffusion-weighted MRI that associate with iron metabolism and protein turnover of venous thrombi showed diagnostic potential in assessing thrombus age and predicting successful thrombus lysis without the need for exogenous contrast agents.^4,17^ However, direct imaging of collagen in DVT, at the molecular level, has not been achieved to date. This information could be valuable in drug development studies to assess the in vivo efficacy of new antifibrotic therapies in preclinical studies. In future translational studies, the ability to detect collagenous thrombus could improve disease stratification and inform therapeutic decisions, such as avoiding thrombolysis in cases of collagenous thrombus, which are unlikely to lyse.

In this study, EP-3533 imaging and statin treatment were used as proof of concept approaches to demonstrate that molecular MRI can feasibly and noninvasively detect changes in collagen content during thrombus resolution. Although EP-3533 is not currently approved for clinical use, advances in medicinal chemistry have been explored to enable safe clinical translation of collagen imaging. To eliminate the risk for gadolinium release, which has been associated with nephrogenic system fibrosis, the linear chelate diethylenetriamine pentaacetic acid used to coordinate gadolinium in EP-3533 has been replaced with the more kinetically inert chelator tetraazacyclododecane tetraacetic acid in CM-101. Compared with EP-3533, CM-101 showed shorter half-life, reduced retention in the liver and kidneys, and increased conspicuity in detecting liver fibrosis.^37^ The imaging window (60–90 minutes post-injection) and the injected dose (10 μmol/kg, which is 100-fold lower than the 0.1 mmol/kg currently used in patients for nontargeted Gd agents) make this approach both suitable and practical for clinical application. Moreover, our in vivo animal experiments were conducted on a clinical scanner with MRI protocols and quantitative T1 mapping sequences used in patients. New accelerated MR image acquisition and reconstruction methods may further minimize the barrier for clinical translation by reducing scan time and cost.^38,39^ Finally, the deployment of clinical low-field MRI scanners (0.55T) may further facilitate the translation of molecular MRI of collagen as the higher *r1* relaxivity of gadolinium agents at lower field strengths, which leads to a stronger signal, may potentially enable reduction of the injected dose.

An alternative route to clinical translation of molecular imaging of collagen is PET. The tracer, ^68^Ga-CBP8, an analogue to EP-3533, has been used to assess lung and cancer fibrosis in preclinical models and is currently undergoing clinical trials.^25,26^ PET imaging probes have a less stringent pathway to human translation, given the low concentrations of the radiolabeled tracer administered, their short half-life, and the comparative ease of probe development compared with gadolinium-based agents.^25^ Advancements in the development of PET and MRI probes are accelerating the clinical translation of molecular imaging, providing a promise in collagen imaging for diagnosing postthrombotic changes.

### Study Limitations

We used the only commercially available formulation of the collagen-specific probe that uses diethylenetriamine pentaacetic acid to coordinate gadolinium. Although, no issues with gadolinium toxicity were observed in animals, future translational MRI studies will require the use of the gadolinium probe, CM-101,^37^ or the recently developed analogue manganese probe, Mn-CBP20.^40^ Future studies in larger animal models may result in prolonged scan times and complicated processing for in vivo R1 analysis. However, faster T1 mapping sequences are now available on clinical scanners, making time-efficient T1 mapping protocols feasible for clinical use.

### Conclusions

Molecular MRI using a collagen-specific imaging probe detects changes in collagen turnover in venous thrombus and enables direct detection of collagenous thrombus. Collagen-targeted MRI also detected the reduction of collagen accumulation after treatment with statins in DVT.

## ARTICLE INFORMATION

### Acknowledgments

The authors acknowledge Dr Tian Yuan (Department of Mechanical Engineering, Imperial College London, London, United Kingdom) for his contribution in writing MATLAB code to process and analyze the magnetic resonance imaging data, Dr Marcelo E. Andia (Radiology Department and Biomedical Imaging Center, Pontificia Universidad Católica de Chile, Santiago, Chile) for his contribution in statistic analysis during revision, and the London Metallomics Facility at King’s College London for their expertise in elemental analysis. The authors would also like to acknowledge the support received from the Great Britain-China Educational Trust Award.

### Sources of Funding

This work has been supported by the King’s British Heart Foundation (BHF) Center of Research Excellence (grant RE/24/130035); (2) BHF Project grant PG/2019/34897; and (3) core funding from the Wellcome Engineering and Physical Sciences Research Council Center for Medical Engineering (grant WT203148/Z/16/Z) and the National Institute for Health and Care Research (NIHR) Clinical Research Facility and HealthTech Research Center in Cardiovascular and Respiratory Medicine at Guy’s and St Thomas’ National Health Service (NHS) Foundation Trust. The views expressed are those of the author(s) and not necessarily those of the NHS, the NIHR, or the Department of Health and Social Care.

### Disclosures

None.

### Supplemental Material

Supplemental Methods

Tables S1–S2

Figures S1–S6

References 41–43

## Supplementary Material
