# Supplementary material for: Molecular MRI of Collagen Enables Evaluation of Fibrosis and Therapeutic Response in Venous Thrombosis
Source: Circ Cardiovasc Imaging. 2025 Dec 10;19(1):e018784. doi: 10.1161/CIRCIMAGING.125.018784 (PMC7618542; doi:10.1161/CIRCIMAGING.125.018784)
Supplement: Supplementary file 1 [file hci-19-e018784-s001.pdf]

SUPPLEMENTAL MATERIAL

Supplemental Methods

Murine model of deep vein thrombosis

Adult 8-10 weeks old male BALB/c mice (weighing 25-30 grams) were purchased from Charles River Laboratories (Margate, United Kingdom) and were randomly assigned to different experimental groups. After 1 week of acclimatization, venous thrombus was induced in the IVC using a surgical procedure combining stenosis of the IVC and endothelial damage. Mice (n=45) were anaesthetised with inhalation of isoflurane at rate of 3-4% and maintained at 2.0-2.5% during surgery. Mice underwent laparotomy to expose the renal vein, inferior vena cava (IVC) and aorta. A gap was then gently created by placing tweezers between the IVC and the adjacent aorta underneath the renal vein. A ligature (Mersilk sutures, 4-0, Ethicon, USA) was place through the gap around the IVC within a piece of prolene suture (polypropylene, 5-0, Ethicon, USA). After ligation, the prolene suture was removed immediately creating an 80-90% stenosis of the IVC. Endothelial damage was also induced using a neurosurgical vascular clamp at the section of the IVC below the tie, for 30 seconds, followed by a rest period of 30 seconds and a second application of the clip downstream from the first application. Peri-operative analgesia (Comfortan, 1mg/ml, 0.06mg/kg) and sterile saline were administrated intraperitoneally, and the muscle and skin were sutured (Vicryl, 4-0, Ethicon, USA). During the recovery period, mice were treated with soft food and hydrogel. Animals were housed in groups of 2–4 or in rare occasions of home-cage aggression separately, in ventilated cages under controlled environmental conditions (12 h of light/ dark cycle, at approximately 21°C and humidity 55 ± 10%).

Imaging collagen remodelling during thrombus organisation and resolution

In vivo molecular MRI of the thrombus was performed at day 2 (n=3), week 1 (days 7-9, n=11), week 2 (days 14-15, n=11), and week 3 (days 20-22, n=13) post-DVT surgery. Statin-treated mice (n=7) were imaged at week 3 (days 20-22) post-surgery. Animals were imaged before and 1 hour after tail vein administration of EP-3533 (10 µmol/kg, EPIX Pharmaceuticals, Lexington, MA, USA). EP-3533 is a gadolinium-based, contrast agent with three Gd-DTPA chelates per molecule targeting type I collagen.[19]

***In vivo* thrombus MRI protocol**

In vivo thrombus imaging was performed using a 3 Tesla MR scanner (Philips Achieva, Philips Healthcare, Best, The Netherlands) equipped with a clinical gradient system (200 mT/m/ms) as previously described.[4,16] Mice were anesthetized with 3-4% isoflurane and maintained using 1.5-2% isoflurane delivered via an anaesthetic tubing while scanning. Oxygen-enriched air was used as a carrier gas. Animals were placed supine on a custom built, single–loop, receiver-only ^{1}H surface coil (Philips, Netherlands, diameter=47 mm). The animals' body temperature was maintained using a water-based, MRI compatible heating system. During the scans, mice were monitored with the use of an MRI-compatible video camera system (Philips Healthcare, Best, The Netherlands). Following, an initial survey scan to identify the mouse anatomy, arterial and venous 2D time of flight sequences (TOF) images were acquired. Arterial TOF was acquired with repetition time (TR)=40ms, echo time (TE)=6.2ms, flip angle=60°, field-of-view (FOV)=35×35×17mm, acquired matrix=116×117, slice thickness=0.5mm, resolution=0.3×0.3mm, reconstructed resolution=0.1×0.1mm, slices=50, averages=2, duration=7.5 minutes, and the venous TOF with: TR=50ms

resulting in a duration of 9 minutes with all other parameters maintained. The maximum intensity projection (MIP) images were used to visualize the abdominal aorta, the renal and iliac bifurcations, the inferior vena cava and the region of flow obstruction corresponding to the thrombus. MIP images were used for planning the subsequent scans and for reference to guide the correlation between MR images and histology. Subsequently, a 2D-Look-Locker (LL) sequence planned perpendicular to the ascending aorta, was used to determine the optimal inversion time (TI) for nulling the blood signal in the following inversion recovery T1-weighted images. LL imaging acquisition parameters were: TR=19ms, TE=8.6ms, flip angle=10°, FOV=30x30mm, acquired matrix=80×78, slice thickness=2mm, resolution=0.4×0.4mm, reconstructed resolution=0.3×0.2mm, and duration=1 minute. Subsequently, a 3D, inversion recovery (IR) T1-weighted segmented gradient recalled echo sequence was acquired before (pre) to visualize thrombus rich in methaemoglobin and 1 hour post EP-3533 to visualise late gadolinium enhancement (LGE) for collagen imaging. Transverse slices were acquired starting just above the renal veins and extending to the union of the two common iliac veins. IR-T1w imaging parameters were: TR/TE=27.4/8.2ms, flip angle=30°, matrix size=400×398, acquired spatial resolution=0.1×0.1×0.5mm, inversion time (TI)= 450ms, slices = 30, signal averages = 2, and duration=13 minutes. Finally, T1 mapping of the thrombus before and after contrast injection was performed using a 3D Look-Locker inversion recovery sequence that employs a non-selective inversion pulse followed by twenty segmented readouts for twenty individual inversion recovery images. For T1 mapping, the ECG was simulated with heart rates set to 240 beats per minute resulting in an RR interval of 250 ms. Imaging parameters were: TR/TE =8.9/4.6ms, flip angle =10°, FOV =36×22×12mm, acquired in-plane resolution

=180×101mm, slice thickness=0.5mm, resolution=0.2×0.2mm, reconstructed resolution=0.2×0.2mm, and duration=21 minutes.

Tissue collection and processing

Immediately after imaging, mice were sacrificed by cardiac puncture under anaesthesia. The IVC, containing the thrombus from immediately above the suture to the bifurcation of the iliac veins, was harvested and pinned on the cork. Tissues were either fixed in 10% neutral buffered formalin (3800601E, Leica, Germany) for 24hrs, dehydrated, and embedded in paraffin for histological analyses (n=3-5/ time point); or snap frozen and embedded in optimal cutting temperature (OCT embedding matrix for frozen sections, 361603E, VWR®) for cryosectioning for laser-ablation inductively-coupled-plasma mass-spectrometry (LA-ICP-MS) (n=3/ time point); or stored at -80 °C for western blotting (n= 3/ time point).

Histology

The entire IVC was divided in nine levels and was sectioned transversely (5-10 μm), starting from the ligature (L1) towards the iliac bifurcation (L9) at 500μm intervals. (**Figure S2**). Sections from each level were stained with Masson's trichrome (H15-1KT, Sigma-Aldrich, Dorset, United Kingdom) and Picrosirius red (ab245887, Abcam, Cambridge, United Kingdom) to provide morphological and compositional (collagen) details of the thrombus and the venous wall following the manufacturer's protocol. Immunohistochemistry (IHC) was used to detect collagen I in the thrombus and venous wall. Cryosections were tempered at room temperature (RT) for 20 minutes, and then fixed in 10% neutral buffered formalin for 15 minutes at RT. Sections were washed in phosphate-buffered saline (PBS) and endogenous peroxidase was

inhibited by 3% H_2O_2 diluted in methanol for 10 minutes. Sections were washed in PBS and unspecific binding was blocked with 5% goat serum in 1% bovine serum albumin (BSA) in PBS for 1 hour at RT. Sections were then incubated with the primary antibody (anti-mouse rabbit polyclonal antibody, ab21286, Abcam; dilution 1:150) for 1hour at RT, washed in 0.05% PBS-Tween 20 (PBST) and then incubated with the secondary antibody (goat anti-rabbit immunoglobulins/HRP, P0448, Dako; dilution 1:600) for 1h at RT. After the incubation, samples were washed in PBST and incubated with DAB kit (SK-4100, Vector Lab, USA) and then washed in the distilled water. Nuclei were counterstained with Gill's haematoxylin (GHS332, Sigma Aldrich) for 1 dip, and slides were washed in running water and then dipped once in 1% acid alcohol. Sections were dehydrated and mounted after staining in preparation of imaging. Microscopy images of Masson's trichrome and Picrosirius red-stained sections were taken with a Leica DMRB microscope (Leica microsystems, Germany). Microscopy images of IHC of collagen I stained sections were taken with a slide scanner (Pannoramic 480 DX Scanner, 3DHistech, Hungary). Computer-assisted colour image analysis (Colour Threshold plug in, ImageJ, NIH, Bethesda, MD, US) was used to selectively visualize and segment the collagen area on Masson's trichrome, Picrosirius red and IHC of collagen I stained sections.

Western blotting

Collagen was extracted from thrombi and venous wall by homogenisation in a sample buffer (8 M urea, 2M thiourea, 3% SDS (w/v), 75 mM DTT, 0.03% bromophenol blue, and 0.05 M Tris–Cl, pH 6.8).[41] The homogenates were centrifuged at 14,000 rpm for 15 minutes at 4°C and the supernatant was collected for protein quantification (Pierce™ BCA Protein Assay Kit, 23225). Samples were boiled at 95°C for 5 minutes

and stored at -20°C. Samples were loaded into an SDS-polyacrylamide gel (7.5%) and run at 125 V for 2 hours. Proteins were transferred onto a polyvinylidine difluoride (PVDF) membrane (Immobilon-P; Millipore Corp) at 30 Voltage for 16 hours at 4°C. The membranes were blocked with 5% skimmed milk for 1 hour at RT and then incubated with a collagen I primary antibody (anti-mouse rabbit polyclonal antibody, ab21286, Abcam; dilution 1:1000) and GAPDH (anti-mouse rabbit polyclonal antibody, ab37168, Abcam; dilution 1:2000) overnight at 4°C. Blots were washed in 0.05% Tris Buffered Saline with Tween 20 (TBST) and then incubated with secondary antibody [Chicken Anti-Rabbit IgG H&L (HRP), ab6829, dilution 1:2000] at RT for 1 hour. After the incubation, blots were washed in TBST and then developed with an enhanced chemiluminescence (ECL) reagent (Bio-rad, 1705061, USA). Blot images were acquired with Invitrogen™ iBright™ FL1500 Imaging System (USA) and analysed with ImageJ (NIH).

Laser-ablation inductively-coupled-plasma mass-spectrometry (LA-ICP-MS)

Frozen samples were cut transversely (10 μm) and LA-ICP-MS was performed with an Iridia 193 nm ArF*excimer-based LA system (Teledyne Photon Machines, Bozeman, MT, USA) equipped with the cobalt long-pulse ablation cell. The LA system was coupled to a Thermo Fisher Scientific iCAPTQ ICP-mass spectrometer (Thermo Fisher Scientific, Waltham, MA, USA) via the Aerosol Rapid Introduction System (ARIS). Imaging was performed on tissues, which were mounted on a four-slide sample holder of the cobalt cell (Teledyne Photon Machines) and image was acquired in fixed dosage mode, a scan speed of 300 mL minute−1 Helium as carrier gas, with a vertical and horizontal spatial resolution of 10 μm. Oxygen was used in the collision reaction chamber and the selected isotope of interest, $^{157}Gd^{16}O$ was chosen to

maximise sensitivity whilst minimising isobaric/polyatomic interferences and increasing signal-to-noise ratio. A series of NIST 612 (National Institute for Standards and Technology, Gaithersburg, MD, USA) standard ablation scans were performed before and after experimental samples to correct for instrumental drift. Quantitative imaging was performed using gelatine micro droplet standards sourced from the Institute of Analytical Chemistry, University of Vienna.[42] Standard gelatine solutions (fish skin gelatine from Sigma-Aldrich; 10% w/w) were made with increasing concentrations (0, 173, 351, 684, 1821, 3455 fg) using a multi-element stock solution and single-element standard solutions purchased from LabKings (Hilversum, The Netherlands). ICP-MS and positional data were reconstructed to generate elemental images using the HDF-based Image Processing software (HDIP, Teledyne Photon Machines Inc., Bozeman, MT, USA). A bespoke in-house pipeline, written in Python (version 4), was used to analyse the reconstructed data and produce comparable elemental images. All data is presented on the same intensity scale for comparability (95% percentile).

MR image analysis

MR images were analysed using Horos (Horos Project, Brooklyn, New York, United States). The filling defect in the IVC, attributable to the presence of thrombus, was used as a measure of thrombus volume. Specifically, transverse MRI slices spanning through the region of the filling defect, resulting in no or low signal as observed on fused MR venography and angiography, were manually segmented on a slice-by-slice basis (**Figure S5**). The thrombus volume (mm^3) was calculated by multiplying the thrombus area measured on individual slices (mm^2) by the slice thickness. The area of the thrombus visible on the pre- and post-contrast enhanced IR-T1w images was

also manually segmented on a slice-by-slice basis. This segmentation was reported as MRI signal area (mm^2). Additionally, the regions of interest traced on pre- and post-contrast IR-T1w images were used to calculate the contrast-to-noise ratio (CNR) and signal-to-noise ratio (SNR) based on the following formulas: CNR= (SI tissue - SI muscle)/ SD noise outside body, SNR= SI tissue/ SD noise outside body. Where SI tissue corresponds to the signal intensity of the tissue of interest (thrombus and venous wall); SI muscle corresponds to the signal intensity of the peri-spinal muscle and standard deviation (SD) was measured outside of the body. T1 and R1 maps were reconstructed offline using an in-house script in developed MATLAB.[43] T1 relaxation times (s) and R1 = relaxation rates (s^{-1}) (1/T1) were calculated by manually segmenting the T1 map regions corresponding to the visually enhanced thrombus observed on the pre and post IR images. To match the pre- and post-contrast MRI images, the distances from the renal vein and the iliac bifurcation to the thrombus were used as internal anatomical landmarks (**Figure S1C**). % ΔCNR, % ΔSNR and % ΔR1 was calculated as: [($Measurement_{post\text{-}contrast}$- $Measurement_{pre\text{-}contrast}$)/ $Measurement_{pre\text{-}contrast}$] *100. To match the *in vivo* MRI and histological images, the distances from the renal vein and the iliac bifurcation to the thrombus were used as internal anatomical landmarks. Imaging data with poor image quality hindering segmentation or incomplete data acquisition because of issues with animal welfare were excluded from the analyses as follows: n=1 at week 1 and n=2 at week 2 for T1 mapping; n=1 at week 3 for IR and n=2 for T1 mapping; and n=1 for statin-treated for IR and T1 mapping.

Supplemental Figures

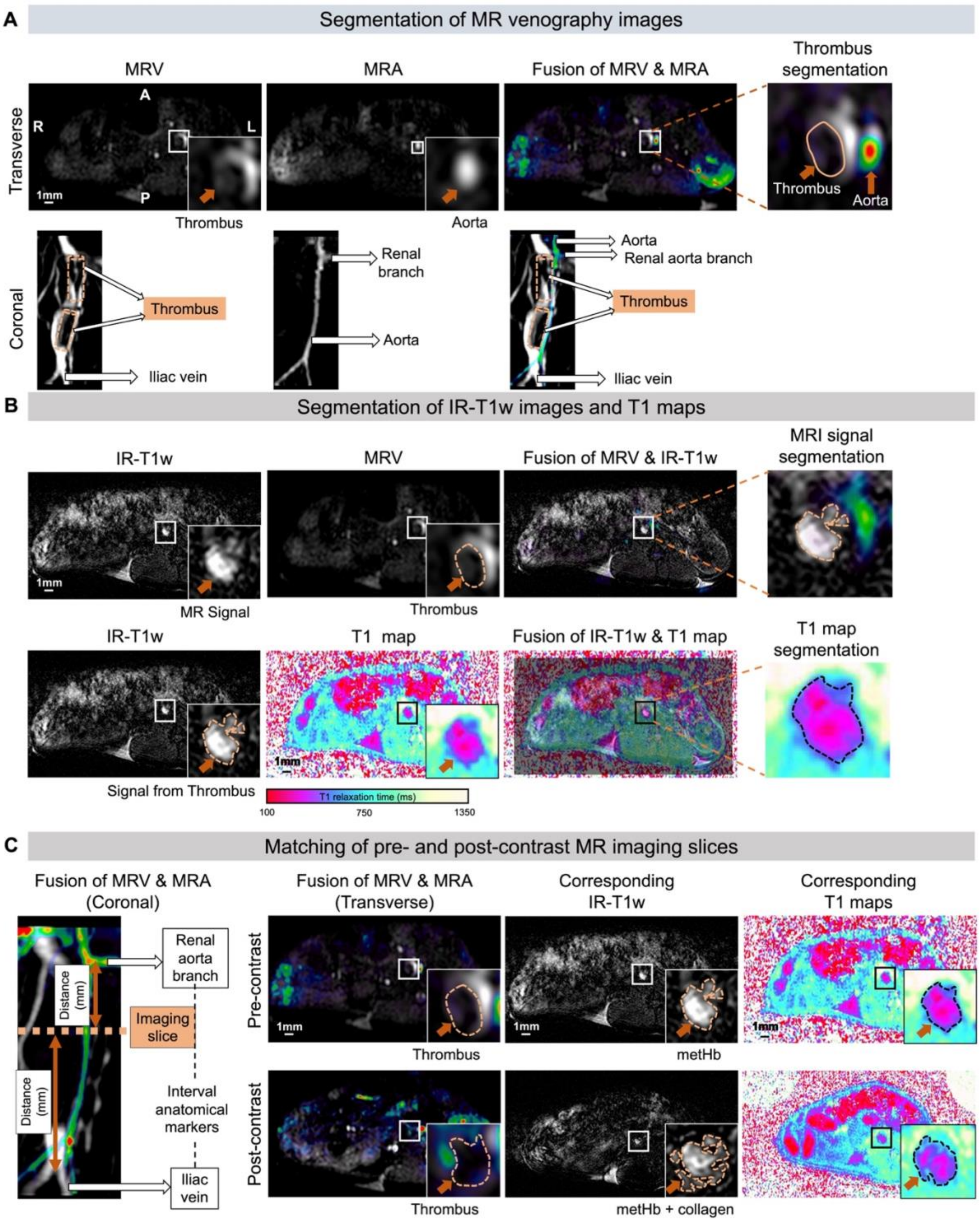

Figure S1. Representative segmentations of the thrombus in MR images. A, Example of segmentation of the thrombus area based on fused MRV and MRA images. **B,** Example of segmentation of the area of the MRI signal observed in the thrombus using fused IR-T1w and MRV images, and T1 maps fused of IR-T1w. **C,** Example of

matching the pre- and post-contrast imaging slice based on the distance from renal aorta branch and iliac vein. MRV: MR venography, MRA: MR angiography. IR-T1W: inversion recovery T1 weighted.

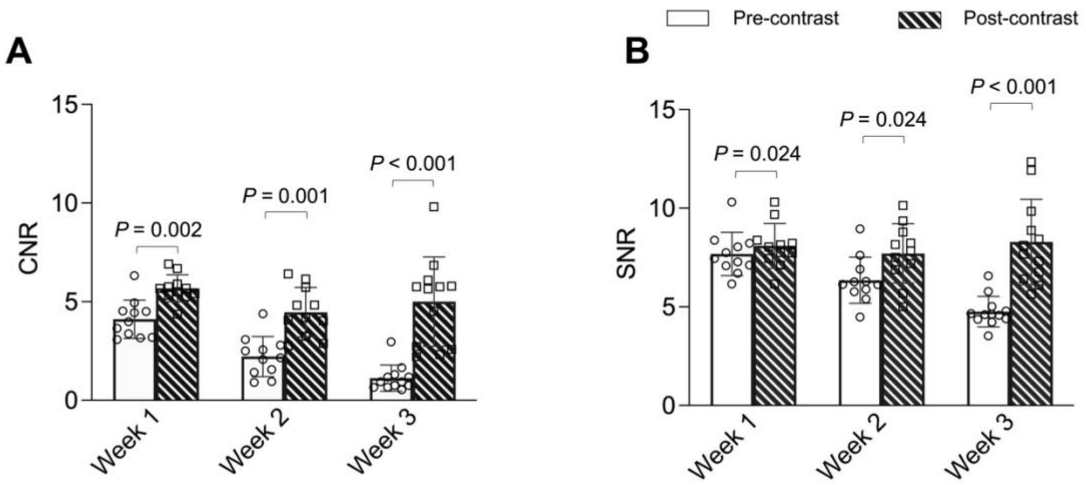

Figure S2. Quantification of signal enhancement pre- and post- administration of the collagen targeting probe. Quantification of CNR **(A)** and SNR **(B)** observed on IR-T1w images. Data are presented as mean±S.D. Wilcoxon matched-pairs signed rank tests for pre- and post-contrast of CNR and SNR, n=11 for week 1, n=11 for week 2, and n=12 for week 3. CNR: contrast to noise ratio, SNR: signal to noise ratio.

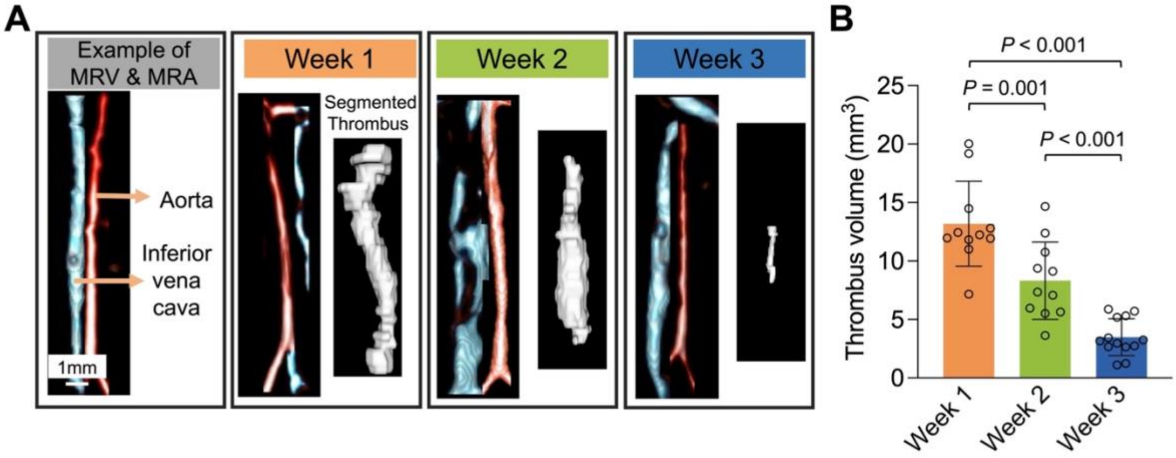

Figure S3. Thrombus resolves overtime in the murine model of DVT. A, Representative MRI showing successfully induced DVT. Fused MR venography (blue) and angiography (red) images detect the inferior vena cava and the abdominal aorta, respectively. Signal voids in the IVC seen at weeks 1, 2 and 3 post-DVT correspond to the thrombus. The volume of the thrombus segmented using the MR venography images is shown in white. **B,** Thrombus volume measured overtime using MR venography. Data are presented as mean±S.D. One-way ANOVA followed Bonferroni's post-hoc test was used multiple group comparisons, n=11 for week 1, n=11 for week 2, and n=13 for week 3 after DVT.

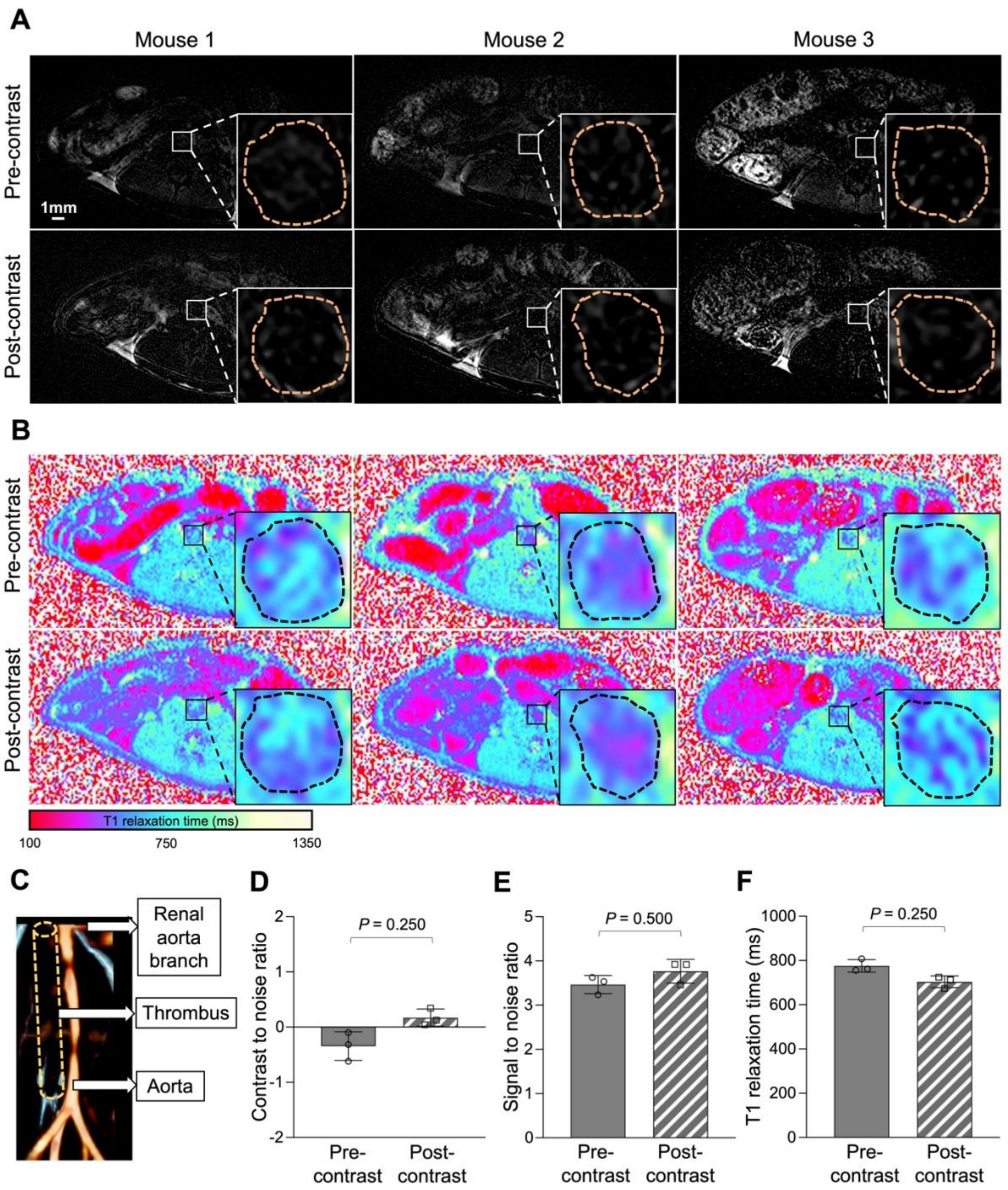

Figure S4. Molecular MRI of collagen showed no unspecific uptake of EP-3533 in acute thrombus at day 2 post-DVT. A, Representative pre- and post-contrast IR-T1w images at day 2 post-DVT. Dashed orange circle line indicates the thrombus. **B,** Corresponding pre- and post-contrast T1 maps at day 2 post-DVT. Dashed orange circle line indicates the thrombus. **C,** Fused MR venography and angiography at day

2 post-DVT. **D-F,** Quantification of pre- and post-contrast CNR, SNR and T1 relaxation time at day 2 post-DVT. Data are presented as mean ± S.D. Wilcoxon matched-pairs signed rank test for CNR, SNR and T1 relaxation time, n=3. IR-T1w: inversion recovery T1 weighted, CNR: contrast to noise ratio, SNR: signal to noise ratio.

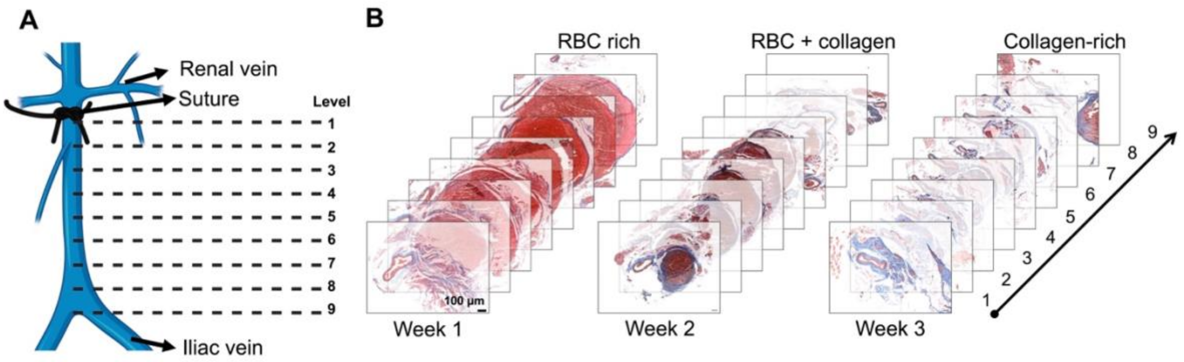

Figure S5. Representative histology images extending from levels 1 to 9 post-DVT. A, Schematic showing the anatomical location of levels 1 to 9 in reference to the ligation and the iliac bifucation. The disctance between each level is 500μm. **B,** Representative Masson's trichrome stain images of the nine levels.

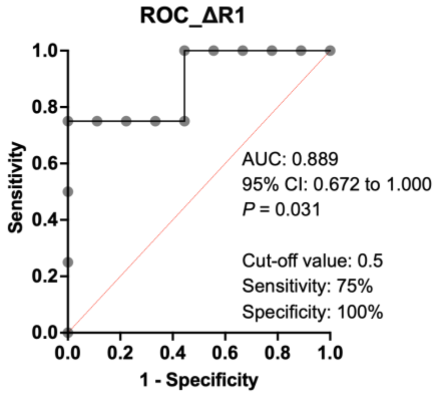

Figure S6. Sensitivity and specificity of molecular MRI in detecting collagen-rich thrombus. ROC curves analysis of ΔR1 from molecular MRI. ROC: receiver operating characteristic. AUC: area under the curve. CI: confidence intervals.

Supplemental Tables

Table S1. Molecular MRI measurements before and after administration of the of collagen

**MRI measurement**	**Time point**	**Pre-contrast**	**Post-contrast**	***P* value**
MRI signal area on IR-T1W images (cm^2)	Week 1	0.14±0.05	0.19±0.08	*P*=0.001
	Week 2	0.05±0.03	0.11±0.04	*P*=0.001
	Week 3	0.02±0.04	0.09±0.06	*P*=0.001
T1 relaxtion time (ms)	Week 1	610±48	524±53	*P*=0.002
	Week 2	732±93	566±75	*P*=0.004
	Week 3	980±157	693±57	*P*=0.002

* *P*-values were calculated using Wilcoxon matched-pairs signed rank test.

Table S2. *Ex vivo* anlysis of collagen quantification

***Ex vivo* technique**	**Week 1**	**Week 2**	**Week 3**	***P* value**
Masson's trichrome staining (mm^3)	0.16±0.09	0.32±0.15	0.55±0.07	*P*<0.001
Picrosirius red staining (mm^3)	0.15±0.09	0.22±0.07	0.52± 0.06	*P*<0.001
Immunohistochemistry of Col I (mm^2)	0.05±0.01	0.09±0.01	0.15±0.01	*P*<0.001
Western blotting (relative expression of Col I)	0.20±0.13	0.60±0.27	1.21±0.52	*P*=0.023

* *P*-values were calculated using a one-way ANOVA followed Bonferroni's post-hoc test.
